# 7 T MRI of macrophages in mouse carotid atherosclerosis using novel nanoparticle platforms

**DOI:** 10.1186/1532-429X-11-S1-P151

**Published:** 2009-01-28

**Authors:** Hisanori Kosuge, Masahiro Terashima, Masaki Uchida, Sarah Sherlock, Philip S Tsao, Mark J Young, Trevor Douglas, Hongjie Dai, Michael V McConnell

**Affiliations:** 1 Cardiovascular Medicine, Stanford University School of Medicine, Stanford, CA USA; 2 Department of Chemistry and Biochemistry, Montana State University, Bozeman, MT USA; 3 Department of Chemistry, Stanford University, Stanford, CA USA

**Keywords:** Left Common Carotid Artery, Left Carotid Artery, Macrophage Accumulation, Protein Cage, Carotid Lesion

## Introduction

Macrophages are important imaging targets for identifying and monitoring high-risk atherosclerotic plaque. Human protein cages and graphite/FeCo core-shell nanocrystals are unique, multi-functional nanoparticle platforms for cellular imaging.

## Purpose

To evaluate 7 T MRI using human ferritin protein cage nanoparticles (HFn) and graphite/FeCo core-shell nanocrystals (CN) for detecting macrophage accumulation in mouse carotid atherosclerosis *in vivo.*

## Methods

A macrophage-rich carotid lesion in FVB mice (N = 6) was induced as follows: high fat diet for 4 weeks then diabetes induction by 5 daily intraperitoneal injections of streptozotocin. Two weeks later, we performed carotid ligation of the left common carotid artery. Either one of two different nanoparticles, HFn or CN, was injected into mice via tail vein 2 weeks after carotid ligation (N = 3 for each nanoparticle).

1) HFn-Fe are human ferritin protein cages chemically modified to contain an iron oxide core (dose – 25 mgFe/kg).

2) CN have a FeCo core with a graphite shell, with Cy5.5 also attached for fluorescence imaging (dose – 32.14 μgFe/mouse, 8 nmol Cy5.5/mouse).

MRI at 24 and 48 hours was performed on 7 T MRI scanner (Varian, Inc. Walnut Creek, CA) using a gradient echo sequence (TR/TE = 50/4.2, slice thickness = 0.5 mm, FOV = 3 cm, matrix = 256 × 256, FA = 50, acquisition time = 9 min 55 sec). Accumulation of nanoparticles on MRI was measured as the size of susceptibility artifacts (% reduction of lumen area). After final *in vivo* MRI, both *in situ* and *ex vivo* fluorescence imaging was performed using Maestro™ *in vivo* imaging system (CRi, Woburn, MA). Perl's iron and immunofluorescence staining was also performed to confirm co-localization of nanoparticles with *macrophages.*

## Results

**Figure 1:**
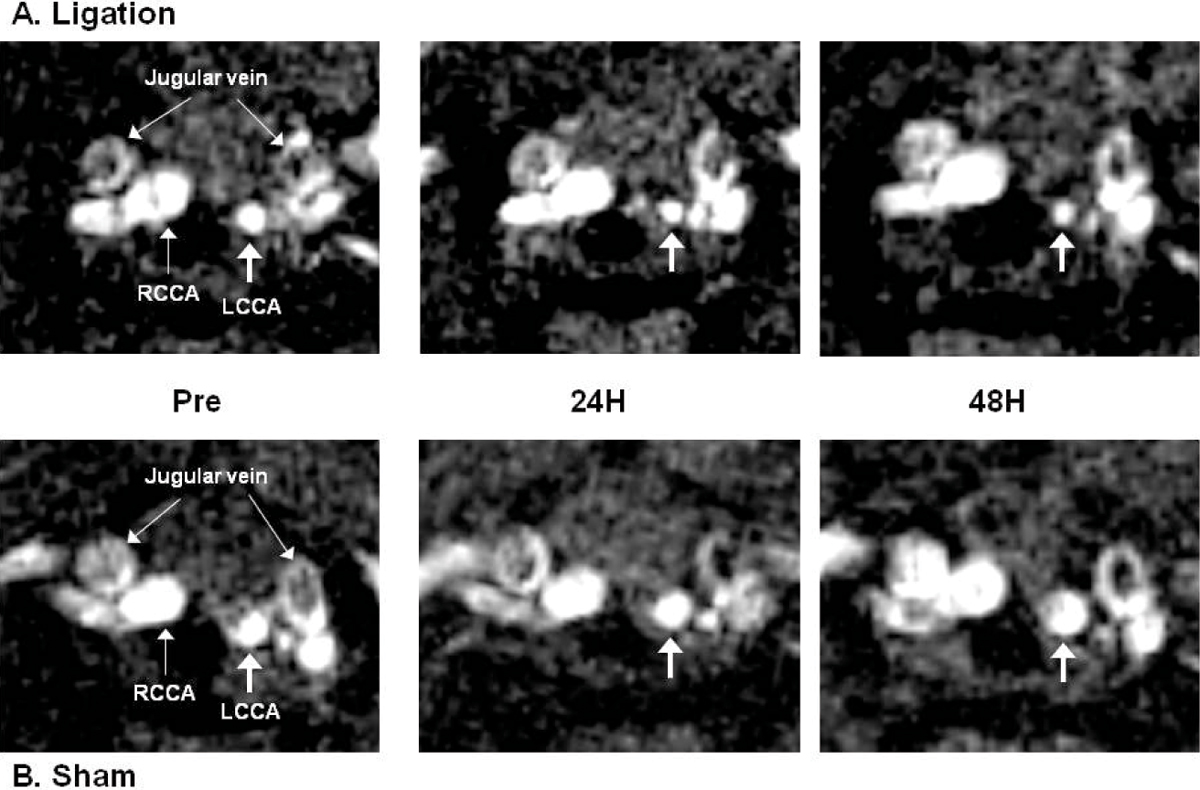
Figure 1

**Figure 2:**
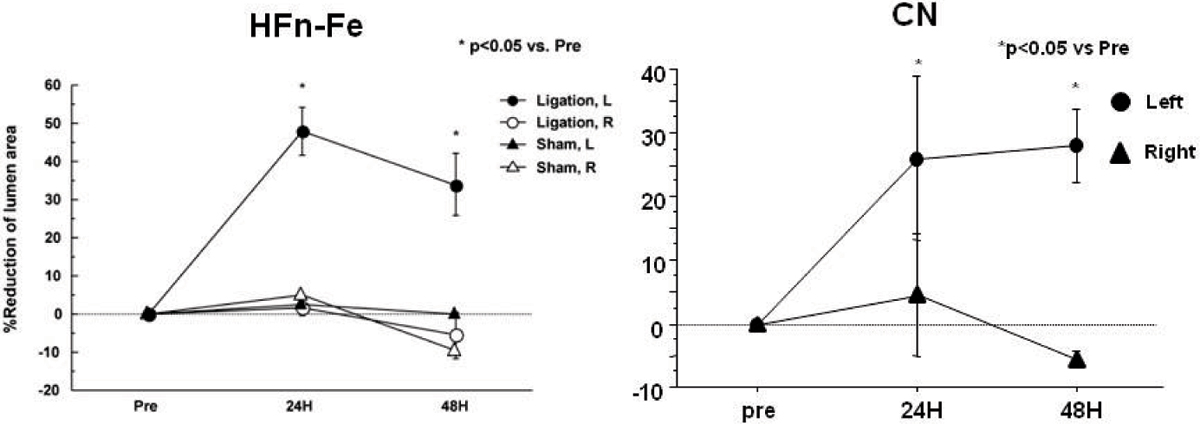
Figure 2

**Figure 3:**
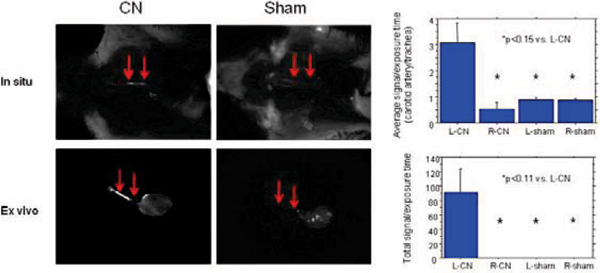
Figure 3

**Figure 4:**
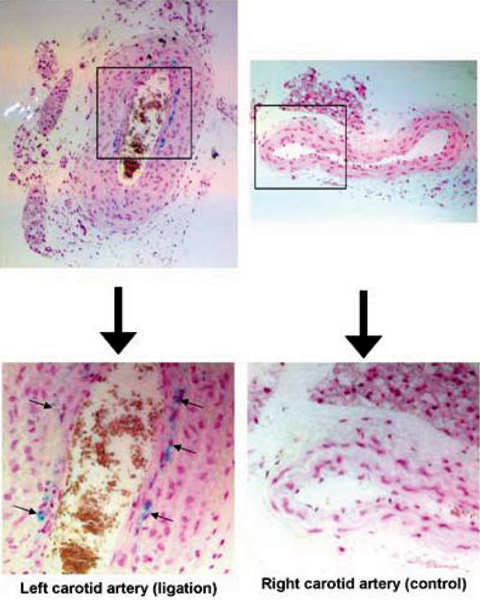
Figure 4

*In vivo* MRI showed the accumulation of HFn-Fe and CN in the ligated left carotid arteries (Figure 1A), with T2* signal loss causing significant reduction in luminal area (HFn-Fe; 24 H 48 ± 8.9%, 48 H 33.9 ± 11.3%, CN; 24 H: 26.1% ± 13.0%; 48 H: 28.0% ± 5.7%, Figure 2). There was no evidence of T2* signal loss in the non-ligated right carotid arteries (HFn-Fe; 24 H: 1.6 ± 2.8%, 48 H: -5.6 ± 8.8%, CN; 24 H: 4.6% ± 9.6%, 48 H: -5.5% ± 1.2%). No significant signal was seen in sham-operated mice (HFn-Fe; 24 H: 2.7% and 48 H: -0.2%, Figure 1B and 2). For confirmation, CN fluorescence imaging showed high signal intensity in the left carotid arteries (Figure 3) with no accumulationseen in either non-ligated right carotid arteries or sham-operated left carotid arteries. HFn-Fe uptake by macrophages was confirmed on histology (Figure 4).

## Conclusion

Both iron oxide protein cage nanoparticles and graphite/FeCo core-shell nanocrystals allow noninvasive MRI of macrophage accumulation in mouse atherosclerosis. These powerful, multi-functional nanoparticles may allow improved noninvasive characterization of vascular inflammation and atherosclerosis.

